# Recent Advances in the Synthesis of Rosettacin

**DOI:** 10.3390/molecules29102176

**Published:** 2024-05-07

**Authors:** Xiao Tang, Yukang Jiang, Liangliang Song, Erik V. Van der Eycken

**Affiliations:** 1 College of Science, Nanjing Forestry University, Nanjing 210037, China; xiaotang@njfu.edu.cn (X.T.);; 2 Jiangsu Provincial Key Lab for the Chemistry and Utilization of Agro-Forest Biomass, Jiangsu Co-Innovation Center of Efficient Processing and Utilization of Forest Resources, Jiangsu Key Lab of Biomass-Based Green Fuels and Chemicals, International Innovation Center for Forest Chemicals and Materials, College of Chemical Engineering, Nanjing Forestry University, Nanjing 210037, China; 3 Laboratory for Organic & Microwave-Assisted Chemistry (LOMAC), Department of Chemistry, University of Leuven (KU Leuven), Celestijnenlaan 200F, B-3001 Leuven, Belgium; 4 Peoples’ Friendship University of Russia (RUDN University), Miklukho-Maklaya Street 6, 117198 Moscow, Russia

**Keywords:** rosettacin, camptothecin, aromathecin, heterocycle, antitumor

## Abstract

Camptothecin and its analogues show important antitumor activity and have been used in clinical studies. However, hydrolysis of lactone in the E ring seriously attenuates the antitumor activity. To change this situation, aromathecin alkaloids are investigated in order to replace camptothecins. Potential antitumor activity has obtained more and more attention from organic and pharmaceutical chemists. As a member of the aromathecin alkaloids, rosettacin has been synthesized via different methods. This review summarizes recent advances in the synthesis of rosettacin.

## 1. Introduction

Compared with all-carbon cycles, heterocycles often own different physical and chemical properties due to the presence of heteroatoms [1,2,3,4,5,6,7]. Thus far, heterocycles are the largest branch of organic compounds. In addition, they are not only important in biology and industry but also have great significance in the operation of human society. Their involvement in multiple fields should be given more attention [1,2,3,4]. The main portion of commercial drugs based on mimicking biologically active natural products is heterocycles. The heterocyclic scaffold is widely present in natural products, drugs, bioactive molecules and functional materials [8,9,10]. In the Comprehensive Medicinal Chemistry database, more than 60% of the compounds possess heterocycles [11]. Therefore, researchers continuously design and produce drugs, insecticides, rodenticides and herbicides with better effects based on natural product models [1,2,3,4]. Heterocycles play an important role in biochemical processes and are also the most typical and important organic compounds in living cells [1,2,3,4]. Meanwhile, heterocycles play important roles in the metabolism of living cells [12,13,14]. Heterocycles have many applications in other fields, such as being used as additives in various industries involving photocopying, cosmetics, solvents, plastics, information storage and antioxidants [1,2,3,4]. Therefore, heterocyclic chemistry occupies a large proportion of organic chemistry. Moreover, heterocyclic chemistry could be ceaselessly employed to synthesize a wide variety of heterocycles, which is a never-ending resource [1,2,3,4]. The numerous combinations of heteroatoms, carbon and hydrogen could give diverse heterocycles bearing various physical, chemical and biological properties [1,2,3,4]. Thus, developing concise and efficient strategies for the construction of diverse heterocycles is highly desired. The synthesis of novel heterocyclic scaffolds has always been needed, providing the platform and opportunity for the discovery of new drugs.

**Scheme 1:**
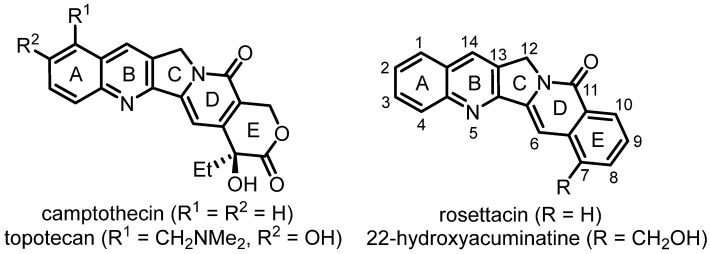
Representative camptothecin and aromathecin alkaloids.

As an important member of heterocyclic scaffolds, indolizino [1,2-*b*]quinolin-9(11*H*)-one constitutes the central moiety of camptothecin (CPT) [15] and aromathecin alkaloids [16] (Scheme 1). CPT was first isolated by Wani and Wall in 1966 from the Chinese tree *Camptotheca accuminata* [17]. In their discovery, the extract exhibited significant anti-tumor activity in in vitro experiments and in mouse leukemia models [18,19]. This result was consistent with the utilization in traditional Chinese medicine as a natural medicine for treating cancer. Meanwhile, some adverse issues with CPT were also observed, such as poor stability and solubility [18,19]. Although the mechanism of action was not clear, CPT was soon approved by the US Food and Drug Administration (FDA) for preliminary clinical trials of colon carcinoma and evaluated as a drug for treating human cancer [18,19]. Although CPT exhibited strong anti-tumor activity in patients with gastric cancer, it also led to serious and unpredictable side-effects such as vomiting, bone marrow suppression, severe hemorrhagic cystic disease and diarrhea [18,19]. These results led to the suspension of the second phase trials in 1972. When further explorations showed that the cellular target of CPT is DNA topoisomerase 1, CPT attracted people’s attention again in the late 1980s [18,19]. Thus far, three compounds in this class (topotecan, belotecan and irinotecan) have been used for clinical treatment of cancer [20]. However, the susceptibility to hydrolysis of the lactone (E ring) generates a hydroxycarboxylate which is inactive and has high affinity for human serum albumin [21]. Thus, more attention has been paid to aromathecin alkaloids, in which the E ring is replaced by a benzene ring [22]. As a member of the aromathecin alkaloids, 22-hydroxyacuminatine is a novel quinoline alkaloid which was isolated along with CPT from the Chinese tree *Camptotheca accuminata* at an extremely low yield of 0.000006% in 1989 [23]. Further biological activity studies indicated that 22-hydroxyacuminatine showed significant cytotoxic activity against the murine leukemia P-388 cells (ED_50_ 1.32 μg/mL) and KB (ED_50_ 0.61 μg/mL) in vitro [23]. Based on these results, more attention has been paid to the efficient synthesis and biological activity investigations of 22-hydroxyacuminatine and its derivatives in order to obtain better anti-tumor activity. Moreover, rosettacin, belonging to the aromathecin alkaloids, has been employed as a CPT/luotonin A hybrid for inhibiting tumor growth by binding to topoisomerase I [24,25,26]. Initial trials showed that the degree of topoisomerase I-dependent DNA cleavage caused by rosettacin was about 50% of that in luotonin A, suggesting that rosettacin was a weak poison [24,25,26]. Rosettacin was also cytotoxic to yeast strains that did not have yeast topoisomerase I but occupied a plasmid containing the human topoisomerase I gene under the control of a galactose promoter [24,25,26]. However, the expression of human topoisomerase I in the yeast strain indeed reduced the cytotoxicity of rosettacin. Further studies showed that 14 substituted rosettacin derivatives owned better antiproliferative activity and anti-topoisomerase I activity than rosettacin, as well as better topoisomerase I inhibitory activity than 22-hydroxyacuminatine [24,25,26]. These derivatives were proposed to undergo the same mechanism with CPT via an intercalation and poisoning process. Aside from the increased solubility and localization to the DNA-enzyme complex, nitrogenous substituents on the 14 position of rosettacin were proposed to be involved in the major groove of the topoisomerase I-DNA complex and possess hydrogen bonds with the amino acids in the major groove, thus stabilizing the ternary complex [24,25,26]. Considering the significant bioactive activity and unique structure bearing a benzo[6,7]indolizino [1,2-*b*]quinolin-11(13*H*)-one core, different strategies have been designed to synthesize rosettacin. These methods not only provide concise and efficient ways to synthesize rosettacin but also give some thoughts on the synthesis of CPT and its analogues as well as other aromathecin alkaloids. This review is focused on the synthesis of rosettacin (Table 1), which is opportune and essential for the rapid growth of this area. This review is organized as a timeline, giving a clear insight for readers to understand the synthesis history of rosettacin.

## 2. Synthesis of Rosettacin

**Scheme 2:**
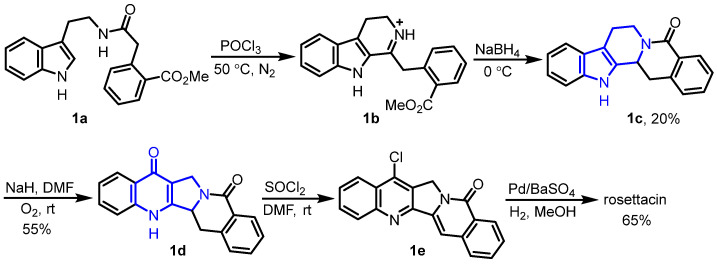
Oxidative rearrangement from indole to quinolone as the key step for the synthesis of rosettacin. Created by us and based on the original work.

In 1972, Warneke and Winterfeldt reported an oxidative rearrangement from indole to quinolone, which was used as the key step for the synthesis of rosettacin [27] (Scheme 2). Firstly, in the presence of POCl_3_, amide **1a** underwent cyclization to form iminium **1b**, followed by sequential NaBH_4_ reduction and intramolecular amidation to give lactam **1c**. By using NaH under O_2_, lactam **1c** underwent oxidative rearrangement, resulting in quinolone **1d**. Finally, quinolone **1d** underwent sequential chlorination using SOCl_2_, followed by aromatization and dechlorination using Pd/BaSO_4_ and H_2_, leading to rosettacin. This approach provided interesting structural skeleton editing for the formation of B and C rings, while the total yield of rosettacin was quite low.

**Scheme 3:**
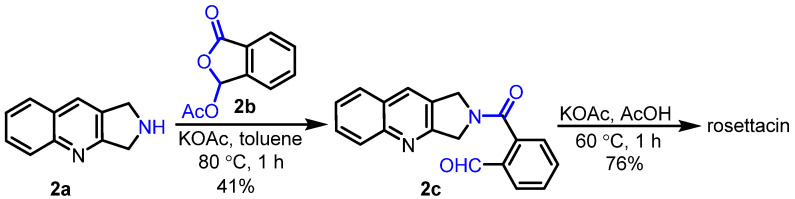
Synthesis of rosettacin from Walraven and Pandit. Created by us and based on the original work.

**Scheme 4:**
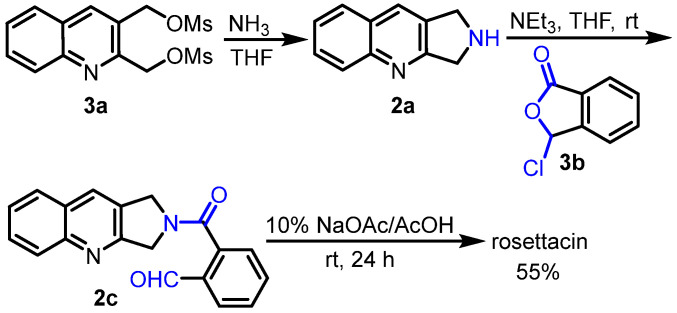
Synthesis of rosettacin from Cushman. Created by us and based on the original work.

In 1980, Walraven and Pandit reported the synthesis of rosettacin [28] (Scheme 3) by learning from Corey’s strategy for the total synthesis of (*S*)-CPT [42]. Dihydropyrroloquinoline **2a** reacted with pseudo-anhydride **2b** under KOAc to give amide **2c**, followed by aldol-type condensation in the presence of KOAc and AcOH to deliver rosettacin. Later on, Cushman et al. employed a similar strategy to synthesize rosettacin [29] (Scheme 4). Dimesylate **3a** reacted with liquid ammonia to afford dihydropyrroloquinoline **2a**, followed by aminolysis of pseudo-anhydride **3b** to form amide **2c**. Under 10% NaOAc/AcOH, intramolecular cyclization of **2c** occurred to produce rosettacin.

**Scheme 5:**
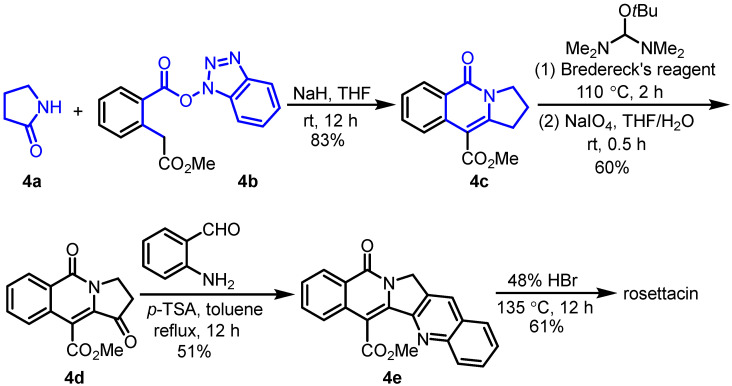
Domino *N*-amidoacylation/aldol-type condensation as the key step for the synthesis of rosettacin. Created by us and based on the original work.

In 2008, Daïch et al. disclosed a form of domino *N*-amidoacylation/aldol-type condensation to generate poly-*N*-heterocycles, which was employed as the key step for the synthesis of rosettacin [30] (Scheme 5). Firstly, lactam **4a** reacted with HOBt ester **4b** in the presence of NaH, resulting in tricyclic product **4c**. When subjected to Bredereck’s reagent [43] at 110 ℃ followed by oxidation using NaIO_4_, keto derivative **4d** was formed. Sequential condensation with 2-aminobenzaldehyde in the presence of *p*-TSA yielded pentacyclic product **4e** according to the Friedländer reaction [44]. Final removal of the ester group upon treatment with 48% HBr at 135 ℃ delivered rosettacin. Although this route was not long, the removal of the ester group required concentrated HBr.

**Scheme 6:**
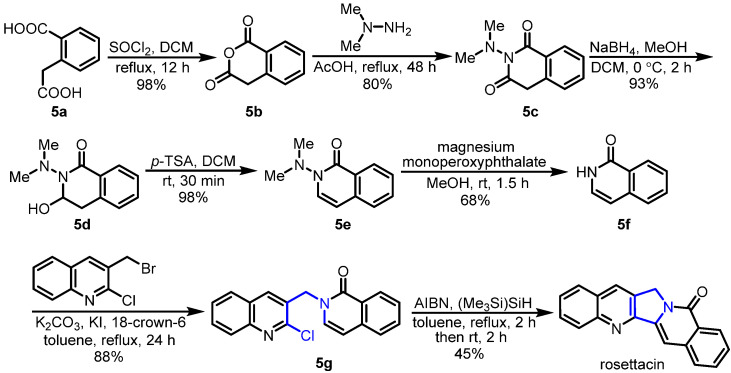
Aryl radical cyclization of enamide as the key ring-closing step for the synthesis of rosettacin. Created by us and based on the original work.

Subsequently, the same group reported another route for the synthesis of rosettacin by using an aryl radical cyclization of enamide as the key ring-closing step [31] (Scheme 6). Firstly, homophthalic acid **5a** reacted with SOCl_2_ under reflux to generate homophthalic anhydride **5b**. Subsequent treatment with *N,N*-dimethylhydrazine in AcOH under reflux resulted in product **5c**. This was followed by NaBH_4_ reduction to afford α-hydroxylactam **5d**. In the presence of *p*-TSA, **5d** underwent dehydration to produce enamide **5e**. Treatment with magnesium monoperoxyphthalate [45] delivered isoquinolin-1(2*H*)-one **5f**. Under phase transfer conditions using K_2_CO_3_, KI and 18-crown-6, *N*-alkylation of **5f** through a reaction with 3-(bromomethyl)-2-chloroquinoline was achieved, affording product **5g**. Sequential treatment with AIBN and (Me_3_Si)_3_SiH via radical cyclization delivered rosettacin. This route required a multistep sequence to isoquinolinone.

**Scheme 7:**
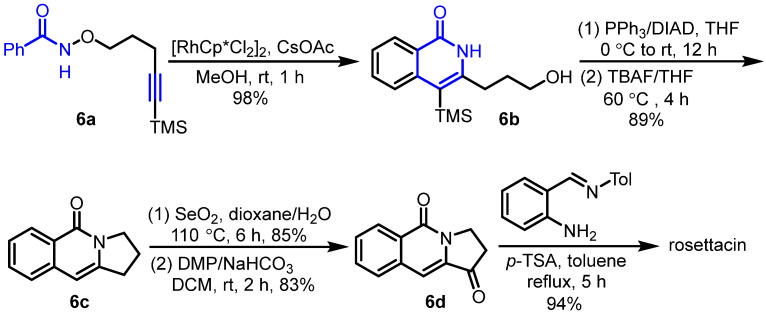
Rh(III)-catalyzed intramolecular C-H activation and annulation of alkyne-tethered hydroxamic ester as the key step for the synthesis of rosettacin. Created by us and based on the original work.

**Scheme 8:**
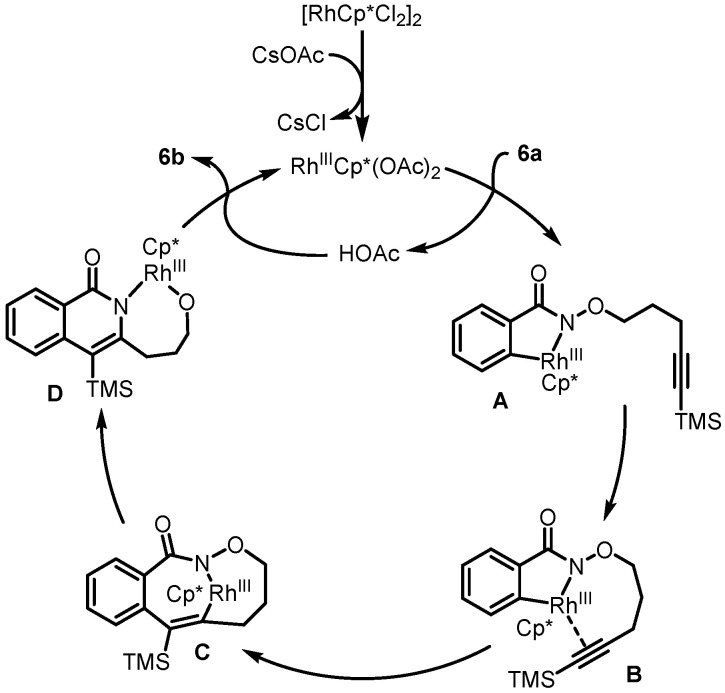
Proposed mechanism for the construction of 3-hydroxyalkyl isoquinolone **6b** from alkyne-tethered hydroxamic ester **6a** via Rh(III)-catalyzed intramolecular C-H activation and annulation. Created by us and based on the original work.

In 2012, Park et al. presented Rh(III)-catalyzed intramolecular C-H activation and annulation of alkyne-tethered hydroxamic esters for the construction of 3-hydroxyalkyl isoquinolones, which served as the key step for the synthesis of rosettacin [32] (Scheme 7). First, hydroxamic ester **6a** bearing a TMS-protected alkyne underwent Rh(III)-catalyzed intramolecular C-H activation and annulation to produce isoquinolone **6b**. This was followed by a Mitsunobu reaction using a PPh_3_/DIAD system and subsequent removal of the TMS group using TBAF to give benzoindolizidine **6c**. Sequential oxidation using SeO_2_ and DMP formed ketone **6d**, which further reacted with *N*-(2-aminobenzylydene)-*p*-toluidine in the presence of *p*-TSA to deliver rosettacin. This method did not tolerate substrates bearing a terminal alkyne, requiring an additional step to remove the TMS group. The mechanism of the generation of product **6b** from substrate **6a** was proposed by the authors (Scheme 8). The dimer [RhCp*Cl_2_]_2_ undergoes the cleavage of the Rh-Cl bond by using CsOAc to afford an active catalyst Rh^III^Cp*(OAc)_2_. An irreversible C-H bond activation of substrate **6a** catalyzed by the Cp*Rh^III^ complex occurs to give a five-member rhodacycle species **A** with the concomitant formation of HOAc. Subsequent coordination of alkyne with the Rh^III^ center produces intermediate **B**. Intermediate **B** undergoes alkyne insertion into the Rh-C bond to afford a seven-member rhodacycle **C**. Sequential reductive elimination and oxidative addition into the N-O bond forms intermediate **D**. Final protonation by HOAc delivers product **6b** and regenerates the active catalyst Rh^III^Cp*(OAc)_2_ for the next cycle.

**Scheme 9:**
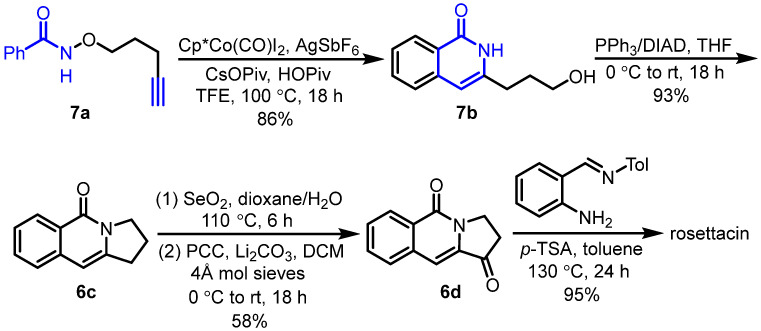
Cp*Co(III)-catalyzed intramolecular C-H activation and annulation of alkyne-tethered hydroxamic ester as the key step for the synthesis of rosettacin. Created by us and based on the original work.

**Scheme 10:**
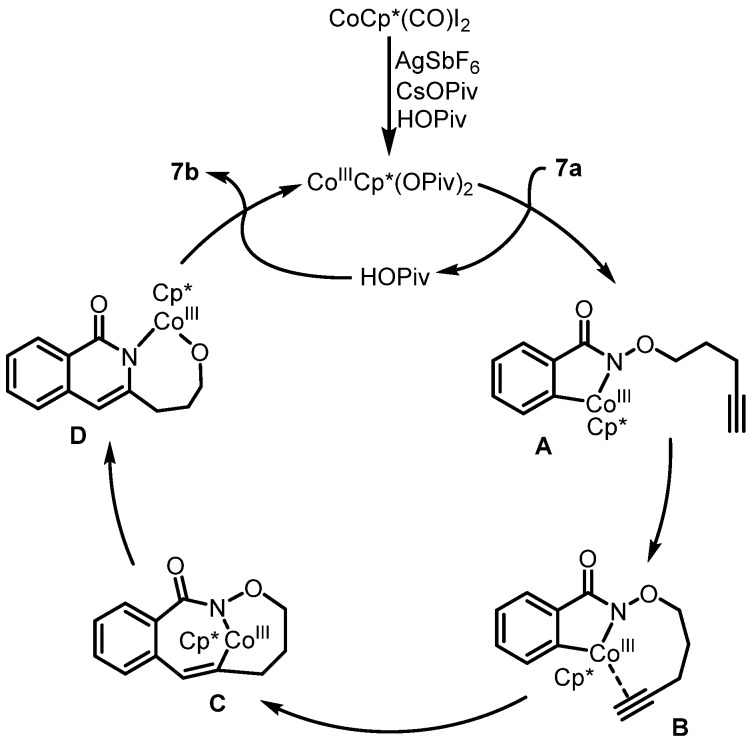
Proposed mechanism for the construction of 3-hydroxyalkyl isoquinolone **7b** from alkyne-tethered hydroxamic ester **7a** via Co(III)-catalyzed intramolecular C-H activation and annulation. Created by us and based on the original work.

Along the same line, Glorius et al. developed Cp*Co(III)-catalyzed intramolecular C-H activation and annulation of alkyne-tethered hydroxamic esters for the construction of 3-hydroxyalkyl isoquinolones, which was used as the key step for the synthesis of rosettacin [33] (Scheme 9). First, hydroxamic ester **7a** bearing a terminal alkyne underwent Cp*Co(III)-catalyzed intramolecular C-H activation and annulation to give isoquinolone **7b**. This was followed by a Mitsunobu reaction using a PPh_3_/DIAD system and sequential oxidation, using SeO_2_ and PCC to generate ketone **6d**. Subsequent treatment with *N*-(2-aminobenzylydene)-*p*-toluidine in the presence of *p*-TSA produced rosettacin. Compared with Rh(III) catalysis, this method not only provided cheap and earth-abundant Co(III) catalysis but also tolerated substrates bearing a terminal alkyne. The mechanism of the generation of product **7b** from substrate **7a** was proposed by the authors (Scheme 10). CoCp*(CO)I_2_ undergoes dehalogenation with silver salt to give the active catalyst Co^III^Cp*(OPiv)_2_. Subsequent C-H bond cleavage of substrate **7a** followed by metalation catalyzed by the Cp*Co^III^ complex generates a five-member cobaltacycle **A** with the concomitant formation of HOPiv. The coordination between alkyne and the Co^III^ center occurs to form intermediate **B**, followed by alkyne insertion into the Co-C bond to produce a seven-member cobaltacycle **C**. Subsequent reductive elimination forms a C-N bond followed by oxidative addition into the N-O bond, which affords intermediate **D**. After protodemetalation of intermediate **D**, product **7b** is released, and the active catalyst Co^III^Cp*(OPiv)_2_ is regenerated for the next cycle.

**Scheme 11:**
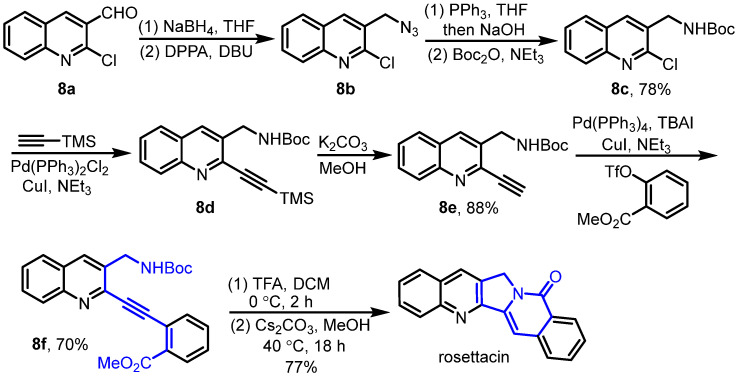
Cascade exo hydroamination followed by spontaneous lactamization as the key step for the synthesis of rosettacin. Created by us and based on the original work.

**Scheme 12:**
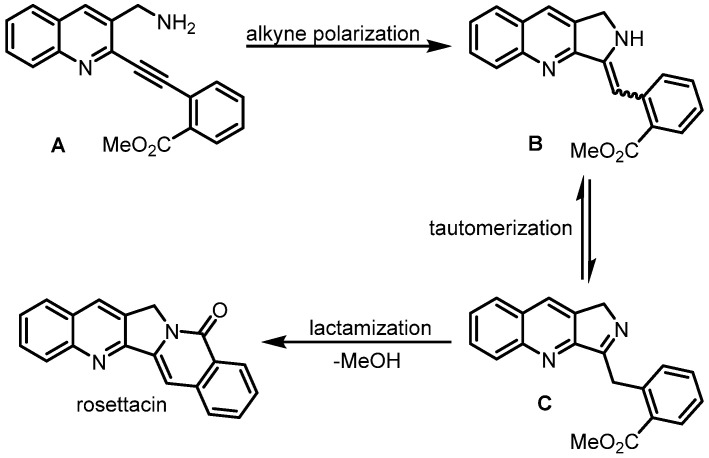
Proposed mechanism for cascade exo hydroamination followed by spontaneous lactamization. Created by us and based on the original work.

In 2016, Gao et al. developed cascade exo hydroamination followed by spontaneous lactamization, which was used as the key step for the synthesis of rosettacin [34] (Scheme 11). Here, 2-Chloroquinoline-3-carbaldehyde **8a** underwent NaBH_4_ reduction, and subsequent azidation using DPPA and DBU yielded azide **8b**. Sequential treatment with PPh_3_ and NaOH formed the primary amine, which reacted with Boc_2_O to afford Boc-protected product **8c**. Subsequent palladium-catalyzed Sonogashira coupling with TMS-protected alkyne and the removal of the TMS group using K_2_CO_3_ generated terminal alkyne **8e**, which underwent a second palladium-catalyzed Sonogashira coupling with ortho ester-substituted trifluoromethanesulfonate to give alkyne **8f**. Treatment with TFA formed the primary amine, followed by adding Cs_2_CO_3_ to deliver rosettacin via exo hydroamination and sequential spontaneous lactamization. Although C and D rings could be formed in one step in this route, the substrate required multistep synthesis. The mechanism of the generation of rosettacin from compound **8f** was proposed by the authors (Scheme 12). Compound **8f** firstly undergoes treatment with TFA to give the primary amine **A**. The alkyne in the primary amine **A** is effectively polarized and activated by the ortho ester-substituted phenyl group, enabling the 5-exo cyclization to form a C-N bond via the primary amine’s addition to a triple bond under basic conditions. This process forms the enamine adduct **B** with a *Z* or *E* geometry, in which the enamine bearing the *Z* geometry favors intramolecular lactamization to deliver rosettacin. For the enamine bearing the *E* geometry, *E*/*Z* isomerization may occur via the formation of imine intermediate **C** to complete the intramolecular lactamization, resulting in the generation of rosettacin.

**Scheme 13:**
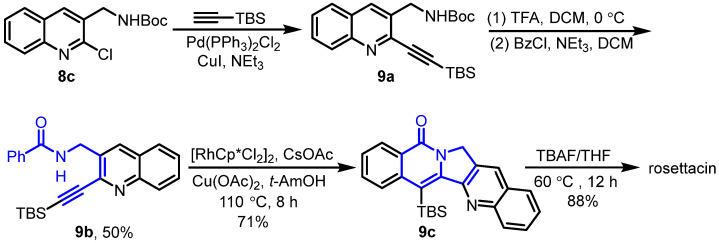
Rh(III)-catalyzed intramolecular C-H activation and annulation of alkyne-tethered benzamide as the key step for the synthesis of rosettacin. Created by us and based on the original work.

**Scheme 14:**
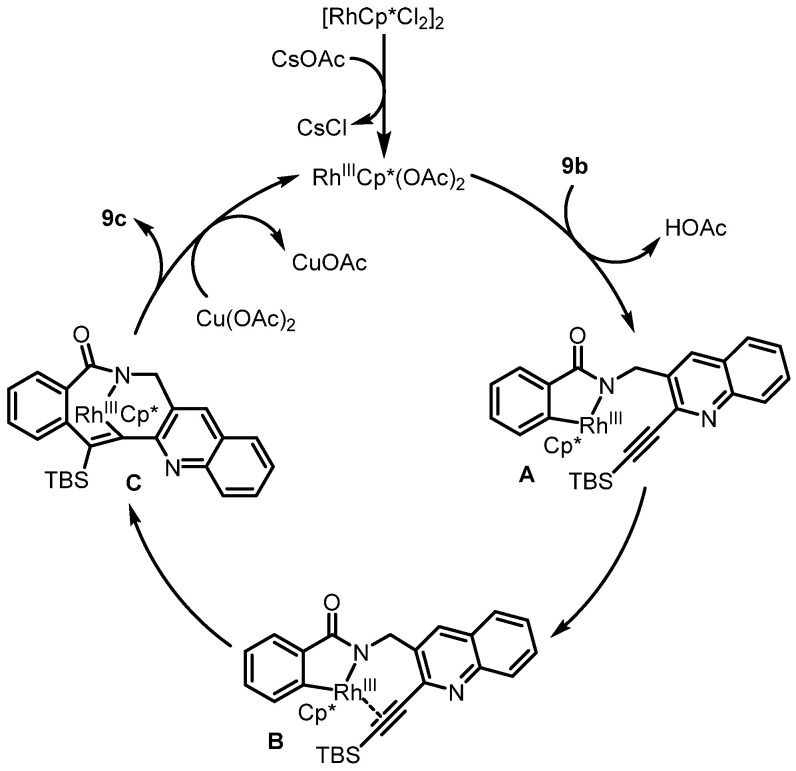
Proposed mechanism for the construction of poly-*N*-heterocycle **9c** from alkyne-tethered benzamide **9b** via Rh(III)-catalyzed intramolecular C-H activation and annulation. Created by us and based on the original work.

In 2017, Van der Eycken et al. reported the Rh(III)-catalyzed intramolecular C-H activation and annulation of alkyne-tethered benzamides for the construction of poly-*N*-heterocycles, which served as the key step for the synthesis of rosettacin [35] (Scheme 13). Quinoline **8c** underwent palladium-catalyzed Sonogashira coupling with TBS-protected alkyne. This was followed by removal of the Boc group with TFA treatment. Sequential amidation with benzoyl chloride gave amide **9b**. Rh(III)-catalyzed intramolecular C-H activation and annulation of the amide **9b** afforded pentacyclic product **9c**, followed by removal of the TBS group under TBAF to deliver rosettacin. This method did not tolerate substrates bearing a terminal alkyne, requiring an additional step to remove the TBS group. The mechanism of the generation of compound **9c** from compound **9b** was proposed by the authors (Scheme 14). The dimer [RhCp*Cl_2_]_2_ undergoes cleavage of the Rh-Cl bond by using CsOAc to afford the active catalyst Rh^III^Cp*(OAc)_2_. Irreversible C-H bond cleavage of compound **9b** catalyzed by the Cp*Rh^III^ complex occurs to give five-member rhodacycle species **A**, with the concomitant formation of HOAc. Subsequent coordination of alkyne with the Rh^III^ center produces intermediate **B**. Intermediate **B** undergoes alkyne insertion into the Rh-C bond to afford seven-member rhodacycle **C**. Sequential reductive elimination delivers product **9c**, followed by regeneration of the active catalyst Rh^III^Cp*(OAc)_2_ from the oxidation of Cp*Rh^I^ by Cu(OAc)_2_.

**Scheme 15:**
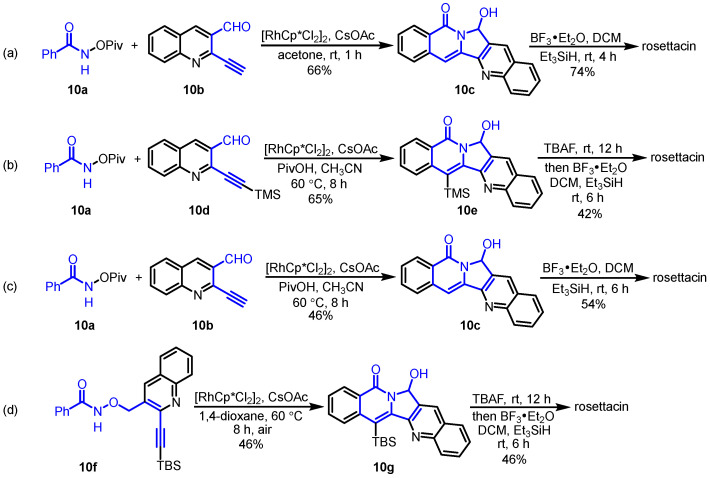
Rh(III)-catalyzed intermolecular C-H activation and annulation of *N*-(pivaloyloxy)benzamide and 2-alkynyl aldehyde as the key step for the synthesis of rosettacin. Created by us and based on the original work.

**Scheme 16:**
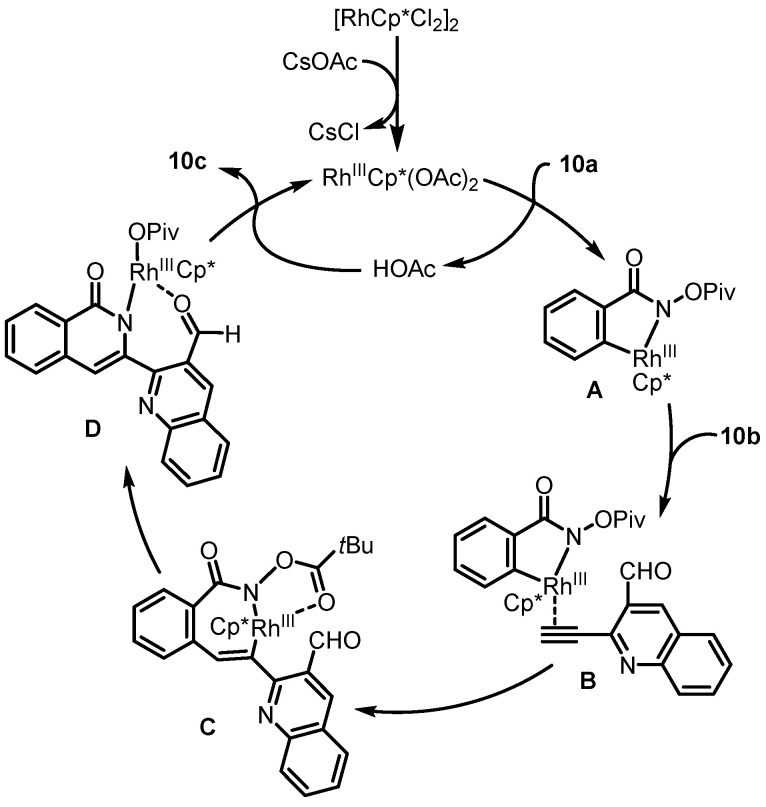
Proposed mechanism for the construction of isoindolo [2,1-*b*]isoquinolin-5(7*H*)-one **10c** from *N*-(pivaloyloxy)benzamide **10a** and 2-alkynyl aldehyde **10b** via Rh(III)-catalyzed intermolecular C-H activation and annulation. Created by us and based on the original work.

In 2018, Reddy and Mallesh disclosed the Rh(III)-catalyzed intermolecular C-H activation and annulation of *N*-(pivaloyloxy)benzamides and 2-alkynyl aldehydes for the construction of isoindolo [2,1-*b*]isoquinolin-5(7*H*)-one, which served as the key step for the synthesis of rosettacin [36] (Scheme 15a). Rh(III)-catalyzed intermolecular C-H activation and annulation of *N*-(pivaloyloxy)benzamide **10a** and 2-alkynyl quinoline-3-carbaldehyde **10b** was performed to yield pentacyclic product **10c**. This was followed by the reduction of aminal using BF_3_•Et_2_O/Et_3_SiH to deliver rosettacin. In the same year, Van der Eycken et al. reported a similar form of Rh(III)-catalyzed intermolecular C-H activation and annulation for the synthesis of rosettacin [37] (Scheme 15b,c). Compared with intramolecular versions, the intermolecular methods avoided multistep sequences to synthesize the substrates, providing a concise and efficient approach to rosettacin. The mechanism of the generation of compound **10c** from substrates **10a** and **10b** was proposed by the authors (Scheme 16). The dimer [RhCp*Cl_2_]_2_ undergoes dehalogenation with CsOAc to yield the active catalyst Rh^III^Cp*(OAc)_2_. Subsequent C-H bond cleavage of substrate **10a** followed by metalation catalyzed by the Cp*Rh^III^ complex generates five-member rhodacycle species **A** with the concomitant formation of HOAc. Coordination between the alkyne in substrate **10b** and the Rh^III^ center in intermediate **A** occurs to form intermediate **B**, followed by alkyne insertion into the Rh-C bond to produce seven-member rhodacycle **C**. Subsequent reductive elimination forming a C-N bond is followed by oxidative addition into the N-O bond, which affords intermediate **D**. After adol-type addition and protonation with HOAc, product **10c** is released, and the active catalyst Rh^III^Cp*(OAc)_2_ is regenerated for the next cycle.

**Scheme 17:**
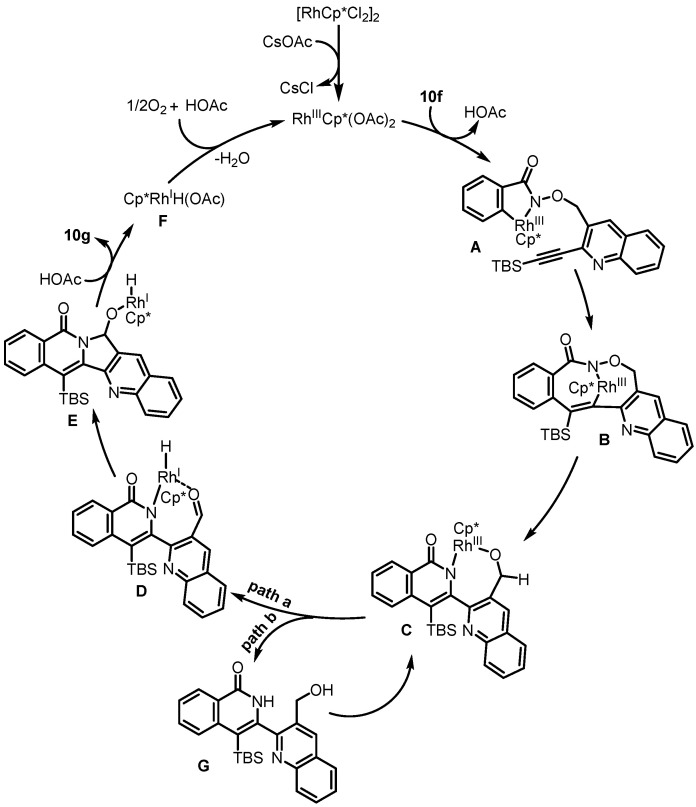
Proposed mechanism for the construction of isoindolo [2,1-*b*]isoquinolin-5(7*H*)-one **10g** from alkyne-tethered hydroxamic ester **10f** via Rh(III)-catalyzed sequential C(sp^2^)-H activation and C(sp^3^)-H amination. Created by us based on the original work.

Based on the above work, Van der Eycken et al. presented Rh(III)-catalyzed sequential C(sp^2^)-H activation and C(sp^3^)-H amination of alkyne-tethered hydroxamic esters, which were employed as the key steps for the synthesis of rosettacin [38] (Scheme 15d). First, alkyne-tethered hydroxamic ester **10f** underwent intramolecular C-H activation and annulation via a rhodium hydride intermediate, resulting in pentacyclic product **10g**, followed by treatment with BF_3_•Et_2_O/Et_3_SiH, at which point rosettacin was formed. This method did not tolerate substrates bearing a terminal alkyne, requiring an additional step to remove the TBS group. Additionally, the alkyne-tethered hydroxamic esters required multistep sequences to prepare. The mechanism of the generation of compound **10g** from substrate **10f** was proposed by the authors (Scheme 17). The dimer [RhCp*Cl_2_]_2_ undergoes cleavage of the Rh-Cl bond by using CsOAc to afford the active catalyst Rh^III^Cp*(OAc)_2_. Irreversible C-H bond cleavage of substrate **10f** catalyzed by the Cp*Rh^III^ complex occurs to give five-member rhodacycle species **A**, with concomitant formation of HOAc. Subsequent coordination of alkyne with the Rh^III^ center and alkyne insertion into the Rh-C bond produces seven-member rhodacycle **B**. Sequential reductive elimination and oxidative addition into the N-O bond forms intermediate **C**. Then, two possible pathways are involved. For path a, intermediate **C** undergoes β-H elimination to yield Cp*Rh^I^ species **D**, followed by adol-type addition of the amide to the aldehyde, forming intermediate **E**. Sequential protonation with HOAc delivers product **10g** and produces Rh-H intermediate **F**. Through an AcOH-O_2_-assisted H_2_O formation process, the active catalyst Rh^III^Cp*(OAc)_2_ can be regenerated from intermediate **F** for the next cycle. For path b, intermediate **C** can undergo protonation to yield intermediate **G**, which would undergo deprotonation to generate intermediate **C** again, and then follow path a to deliver product **10g**.

**Scheme 18:**
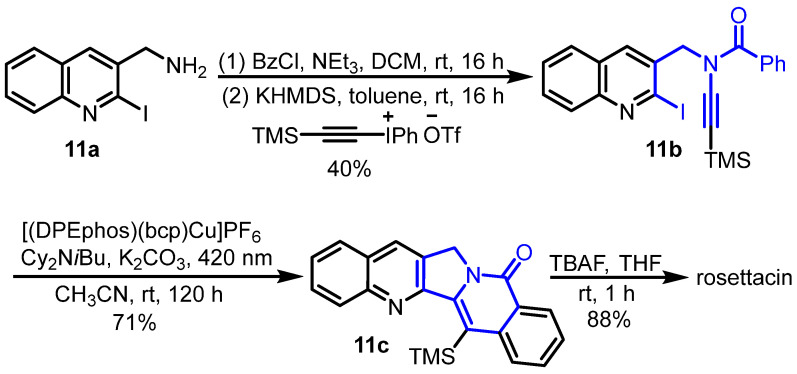
Copper-catalyzed photoinduced radical domino cyclization of ynamide as the key step for the synthesis of rosettacin. Created by us and based on the original work.

**Scheme 19:**
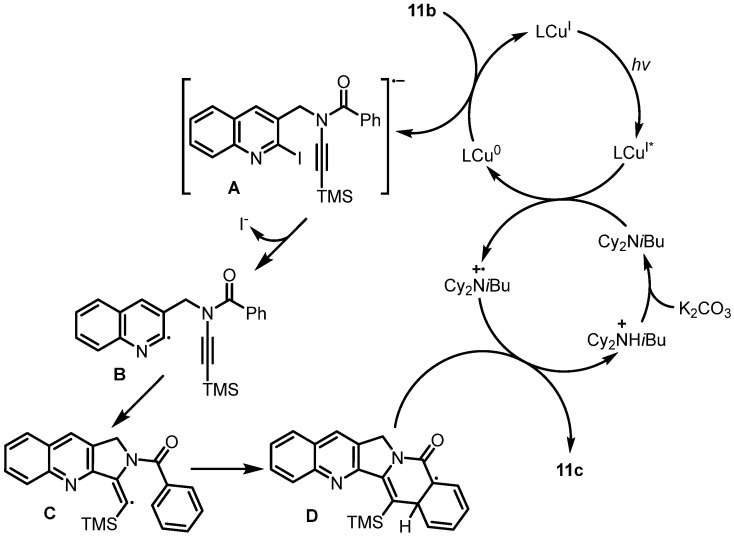
Proposed mechanism for the construction of poly-*N*-heterocycle **11c** from ynamide **11b** via copper-catalyzed photoinduced radical domino cyclization. Created by us and based on the original work.

Additionally, Evano et al. reported the copper-catalyzed photoinduced radical domino cyclization of ynamides for the construction of poly-*N*-heterocycles, which was used as the key step for the synthesis of rosettacin [39] (Scheme 18). Here, 2-Iodo-3-aminomethylquinoline **11a** underwent benzoylation with benzoyl chloride. Subsequent alkynylation using Witulski’s method [46] yielded *N*-benzoylynamine **11b**. Subsequent copper-catalyzed photoinduced radical cyclization generated pentacyclic product **11c**, followed by the removal of the TMS group under TBAF to deliver rosettacin. In this route, copper-catalyzed photoinduced cyclization provided a mild and green method to construct the C and D rings. The mechanism of the generation of compound **11c** from compound **11b** was proposed by the authors (Scheme 19). The ground state LCu^I^ photocatalyst is excited by visible light, followed by single-electron transfer (SET) with the tertiary amine to yield LCu^0^ species and amine radical cation. The subsequent SET between compound **11b** and LCu^0^ affords radical anion **A** and regenerates the ground state LCu^I^ for the next cycle. After deiodination of intermediate **A**, radical intermediate **B** is formed, followed by radical addition to the triple bond via 5-exo-dig cyclization, at which point vinylic radical intermediate **C** is generated. Intermediate **C** undergoes radical cyclization to produce intermediate **D** via a 6-endo-trig process, followed by aromatization via hydrogen atom transfer (HAT) with amine radical cation to deliver product **11c** and amine salt. Under K_2_CO_3_, the tertiary amine is regenerated from the amine salt for the next cycle.

**Scheme 20:**
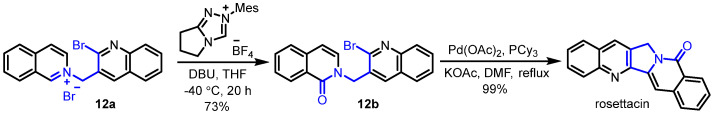
Carbene-catalyzed aerobic oxidation of isoquinolinium salt as the key step for the synthesis of rosettacin. Created by us and based on the original work.

**Scheme 21:**
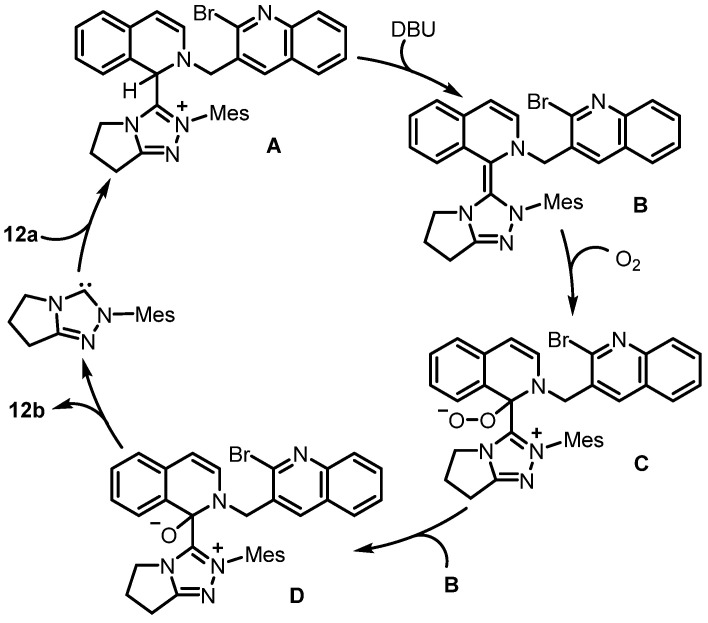
Proposed mechanism for the construction of isoquinolinone **12b** from isoquinolinium salt **12a** via carbene-catalyzed aerobic oxidation. Created by us and based on the original work.

Later on, Fu and Huang developed a form of carbene-catalyzed aerobic oxidation of isoquinolinium salts for the construction of isoquinolinones, which was employed as the key step for the synthesis of rosettacin [40] (Scheme 20). First, the isoquinolinium salt **12a** underwent carbene-catalyzed aerobic oxidation to form isoquinolinone **12b**. Subsequent palladium-catalyzed intramolecular cyclization of **12b** delivered rosettacin. This method provided a metal-free approach to constructing the isoquinolinone scaffold which avoided the employment of transition metals. The mechanism of the generation of compound **12b** from substrate **12a** was proposed by the authors (Scheme 21). The addition of NHC to substrate **12a** yields intermediate **A**, followed by deprotonation under DBU to generate aza-Breslow intermediate **B**. Single-electron transfer (SET) between intermediate **B** and O_2_ followed by radical recombination affords intermediate **C**. Intermediate **C** undergoes interaction with another intermediate **B** to produce intermediate **D**, in which intermediate **B** may serve as the reducing reagent. The final formation of product **12b** from intermediate **D** occurs, regenerating the free NHC for the next cycle.

**Scheme 22:**
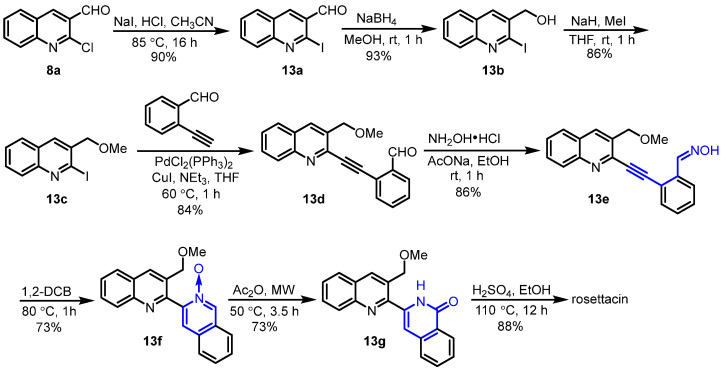
Thermal cyclization and Reissert-Henze-type reaction as the key step for the synthesis of rosettacin. Created by us and based on the original work.

In 2023, Choshi et al. reported a new method for the synthesis of rosettacin by using thermal cyclization and a Reissert–Henze-type reaction as the key step [41] (Scheme 22). Here, 2-Chloroquinoline-3-carbaldehyde **8a** reacted with NaI and concentrated HCl to yield 2-iodoquinoline **13a**. Sequential NaBH_4_ reduction and methylation using MeI afforded 3-methoxymethyquinoline **13c**. Palladium-catalyzed Sonogashira coupling with 2-ethynylbenzaldehyde followed by treatment with hydroxylamine produced oxime **13e**. Oxime **13e** underwent thermal cyclization in 1,2-DCB to form *N*-oxide **13f**, followed by a Reissert–Henze-type reaction using Ac_2_O and microwave conditions to afford isoquinolone **13g**. Heating with H_2_SO_4_ in EtOH transformed the isoquinolone **13g** into rosettacin. This route required a multistep sequence to construct the C/D rings.

## 3. Conclusions

Heterocycles are a significant kind of organic compound in synthetic chemistry. Heterocyclic scaffolds widely exist in many natural products, drugs, bioactive molecules and functional materials. Due to their versatile bioactive activities and functions, heterocycles not only play vital roles in biology and industry but also serve as important building blocks for an array of useful transformations. As a representative member of heterocycles, camptothecin (CPT) has been isolated from the Chinese tree *Camptotheca accuminata* and shows significant anti-tumor activity. Unfortunately, poor solubility and stability as well as unpredictable adverse drug–drug interactions limit its development. A wide range of structural modifications of CPT and the corresponding anti-tumor activity tests produce three compounds (topotecan, belotecan and irinotecan), which have been employed for the clinical treatment of cancer. However, CPT and its analogues face inherent structural problems. The susceptibility to hydrolysis of the lactone in the E ring generates a hydroxycarboxylate, which is inactive and has high affinity for human serum albumin. This seriously reduces antitumor activity. To change this situation, aromathecin alkaloids were investigated in order to replace camptothecins with them.

Rosettacin, belonging to the aromathecin family, has attracted the attention of organic and pharmaceutical chemists. Considering its important bioactive activity and unique structure, an array of strategies have been developed for the synthesis of rosettacin. For example, oxidative rearrangement from indole to quinolone occurs to yield a pentacyclic core. Aminolysis using pseudo-anhydride is performed to afford a tricyclic scaffold. A domino *N*-amidoacylation/aldol-type condensation process is used to form a tricyclic structure. Aryl radical cyclization of enamide is employed as the key ring-closing step for the construction of rosettacin. Rh(III)-catalyzed or Co(III)-catalyzed intramolecular C-H activation and annulation are used for the synthesis of isoquinolones. Cascade exo hydroamination followed by spontaneous lactamization is utilized as the key ring-closing step for the synthesis of rosettacin. Rh(III)-catalyzed intramolecular or intermolecular C-H activation and annulation are used as the key ring-closing step for the synthesis of a pentacyclic core. Copper-catalyzed photoinduced radical domino cyclization of ynamides serves as the key ring-closing step for constructing a pentacyclic core. Carbene-catalyzed aerobic oxidation of isoquinolinium salts is used for the construction of isoquinolinones. Thermal cyclization and a Reissert–Henze-type reaction are used for the synthesis of isoquinolones. These strategies not only provide a platform for the efficient preparation of rosettacin and its analogues but also bring some directions for the synthesis of CPT and its analogues, as well as other aromathecin alkaloids. This is essential for pharmaceutical chemists studying its antitumor activity. They are also beneficial for the discovery of new anticancer drugs. This review summarizes recent advances in the synthesis of rosettacin, which is timely and desirable for the rapid development of this field. Despite major advances, greener as well as more concise and efficient routes are still in demand. We hope this review will help researchers find hidden opportunities and stimulate the development of novel and concise routes for the synthesis of rosettacin.

## Figures and Tables

**Table 1:** A summary on the synthesis of rosettacin.

Year	Author	Key Step	Formed Ring	Reference
1972	Warneke and Winterfeldt	Oxidative rearrangement	B and C	[27]
1980	Walraven and Pandit	Aminolysis and aldol condensation	D	[28]
2003	Cushman	Aminolysis and aldol condensation	D	[29]
2008	Daïch	*N*-Amidoacylation/aldol condensation	D	[30]
2015	Daïch	Aryl radical cyclization	C	[31]
2012	Park	Rh(III)-catalyzed C-H activation	D	[32]
2017	Glorius	Co(III)-catalyzed C-H activation	D	[33]
2016	Gao	*exo* Hydroamination and lactamization	C and D	[34]
2017	Van der Eycken	Rh(III)-catalyzed C-H activation	C and D	[35]
2018	Reddy	Rh(III)-catalyzed C-H activation	C and D	[36]
2018	Van der Eycken	Rh(III)-catalyzed C-H activation	C and D	[37]
2019	Van der Eycken	Rh(III)-catalyzed C-H activation	C and D	[38]
2018	Evano	Cu-catalyzed photoinduced radical domino cyclization	C and D	[39]
2018	Fu and Huang	Carbene-catalyzed aerobic oxidation and Pd-catalyzed cyclization	C	[40]
2023	Choshi	Thermal cyclization and Reissert–Henze-type reaction	D	[41]

## Data Availability

No new data were created or analyzed in this study. Data sharing is not applicable to this article.
